# A bibliometric review of cryptocurrencies: how have they grown?

**DOI:** 10.1186/s40854-021-00306-5

**Published:** 2022-01-01

**Authors:** Francisco Javier García-Corral, José Antonio Cordero-García, Jaime de Pablo-Valenciano, Juan Uribe-Toril

**Affiliations:** 1 Research Group: Almeria Group of Applied Economy (SEJ 147), University of Almeria, Carretera Sacramento s/n, 04120, La Cañada de San Urbano, Almería, Spain; 2 Department of Law, Financial and Tax Law, University of Almeria, Carretera Sacramento s/n, 04120, La Cañada de San Urbano, Almería, Spain; 3 Department of Business and Economics, Applied Economic Area, University of Almería, Carretera Sacramento s/n, 04120, La Cañada de San Urbano, Almería, Spain

**Keywords:** Cryptocurrency, Bitcoin, Ethereum, Bibliometric analysis, Business and economics

## Abstract

With the development of new technologies, some concepts become relevant in the economic area, as is the case with cryptocurrencies, in general, or Bitcoin and Ethereum, in particular. Due to the impact of these tools, a detailed bibliometric study that allows us to obtain all information about cryptocurrencies must be conducted. This study will help scientific production by specifying the development and lines of related research that have been followed and are currently being followed. We have used Tableau, R (Bibliometrix R Package), and VOSviewer software to analyze the information. These have been combined to create and review unified metadata from the Web of Science (WoS) and Scopus databases. The bibliometric analysis shows 771 articles on the WoS database and 648 articles on Scopus published between 2010 and early 2019. They present the most relevant articles, research areas, countries, institutions, authors, journals, and trends during the last few years. In conclusion, the number of publications has grown in the last 3 years. The analysis shows the evolution of blockchain technology used in this type of cryptocurrency. The review of this period marks a possible end to the historical part of cryptocurrencies, thereby opening the current topic to its multiple applications.

## Introduction

In the last decade, secondary payment methods other than legal tender have been developed to boost the market (Corrons 2017). Lietaer and Hallsmith (2006) defined one of these payment mechanisms as an agreement to use more than just legal tender as a means of exchange to link unused sources to unmet needs. In particular, a series of complementary currencies incorporated into the economic world are mentioned. Although these new supplementary payment methods are not listed in any global database, more than 6000 types are presumed to exist. Among them, new electronic payment methods have recently been incorporated, including virtual currencies or cryptocurrencies. Although complementary currencies have been used for a longer period, by historical amount and weight, the central focus of this study is the most innovative cryptocurrencies.

A broad spectrum of terminology are coined to differentiate between these cryptocurrencies, ranging from virtual complementary currency to electronic currency and its derivative, cryptocurrency (Dai 1998). The first currency to become popular was Bitcoin, which was founded in 2008 by Satoshi Nakamoto. Although previous attempts at virtual currencies, such as E-gold in 1996 or Liberty Reverse in 2006, have been made, Bitcoin was the first to exist in the global socio-economic sphere (Garcia et al. 2014).

During these cryptocurrencies’ short period of existence, they have been and are studied by a wide variety of disciplines, as they incorporate a number of innovative technologies, such as blockchain, cryptography, and smart contracts (Xu et al. 2019). Several studies have characterized cryptocurrencies as having a volatile future (Urquhart 2016; Katsiampa 2017; Chu et al. 2017; Conrad et al. 2018; Bouri et al. 2019) and initially presented them as non-perishable albeit secure. However, these promising technologies have kept them (Zheng et al. 2018; Zulfiqar and Gulzar 2021). The globalization process to which they are subjected, together with the lack of legal regulation, indicate that they have been used in multiple forms as the primary component (Gomá-Garcés 2014; Zimmer 2017). They are also the subject of much discussion and debate by entities, such as the European Central Bank (2012) seeking to better define them as a means of exchange and a unit of value accepted by a virtual community.

This article aims to contribute to the extant literature by conducting a bibliometric analysis of the main currencies, as the number of publications on this subject is increasing. Therefore, a review of the materials published in this interdisciplinary area must be incorporated. Moreover, this methodology is applied in multiple areas of knowledge from the mapping analysis of bibliographic information obtained from high-impact databases.

First, we will focus on Bitcoin and Ethereum as the main currencies and the concept of cryptocurrency. The results obtained are intended to inform about a specific field of study and its evolution and productivity. In addition, they help identify, analyze, and organize the main elements of the search focus to show the evolution of trends in the subject. Finally, the results seek to establish whether major changes occur in the lines of research to determine whether the theoretical part is more irrelevant. In this case, the new lines of research will be more practical, changing their orientation and making the previous publications more historical-theoretical.

This method has been used in several studies with similar themes. However, unlike previous studies (Table 1), the present study considered three keywords, along with a new temporal division in the discussion. Exclusively and to increase the importance of this article, this study will include the results of “cryptocurrency, Bitcoin and Ethereum,” thus covering a broader index of results with economic topics from the Web of Science (WoS) and Scopus databases. This differentiates it from the works related to blockchain only as a concept that does not come into discussion, from those that analyze Bitcoin only (e.g., Merediz-Sola et al. 2019; Orastean et al. 2019; Shen et al. 2020), or those that only examine one database (Dabbagh et al. 2019).

**Table 1:** Comparison with previous studies

Diferences		This Paper	A bibliometric analysis of bitcoin scientific production. Merediz-Sola et al. (2019)	Bitcoin In The Scientific Literature—a Bibliometric Study Orastean et al. (2019)	Research development of Bitcoin: a network and concept linking analysis. Shen et al. (2020)	The Evolution of Blockchain: a Bibliometric Study. Dabbagh et al. (2019)
Data base	WoS	*	*	*		*
	Scopus	*			*	
Keywords	Bitcoin	*	*	*	*	*
	Ethereum	*				*
	Cryptocurrency	*				*
Documents and citation references		*	*	*	*	*
Research Area		*	*	*	*	*
Country		*	*	*		
Institutions		*				*
Journals		*	*	*		*
Authors		*	*	*	*	
Keyword Trends		*	*	*	*	
Discussion		*				*

*Source*: Own compilation

This study begins with an introduction and a literature review on alternative forms of payment and their different concepts and interpretations. Then, it explains which selected payment systems have the greatest impact. The methodology of the bibliometric analysis and the sources used to extract the data during the search process are then presented. Subsequently, the results are presented independently, followed by a discussion on the future of these tools with more up-to-date data until 2020. Finally, the last section concludes with both definitive comments and potential research streams from the data analysis.

## Background

### The concept

Cryptocurrencies are a form of digital exchange that ensures that transactions are made through a robust encryption process, which, in turn, controls the number of stocks (Luu et al. 2016). This is a recent phenomenon gaining momentum in a volatile and fluctuating economic world (Ciaian et al. 2016) and has experienced significant growth, despite not being considered an official form of debt cancellation (Dwyer 2015). Due to the decentralized nature of cryptocurrencies, they cannot be used as a substitute for legal currency (Nakamoto 2008) even if they were created to be used as such, thus making them an unconventional currency. The creation and management of currencies are controlled by non-governmental entities (Kim 2015); hence, although they are considered a promising alternative for the future, they have various detractors who prefer to use them as a form of speculation (Baur et al. 2018; Krugman 2018; Zhang et al. 2021). The decentralized structure without regulated activity makes them a novel option to the traditional financial system (Franco 2014). Thus, although they start from a totally negative configuration, they have a series of advantages: cheaper transaction costs due to the absence of intermediaries; reduction of transaction times as these are carried out via the Internet; the suppression of intermediaries as unnecessary financial agents in this series of transactions; or their globality (Kostakis and Giotitsas 2014; Koblitz and Menezes 2016).

In addition, individuals have freedom to develop this type of currency; consequently, multiple currencies have been created for specific purposes (Kondor et al. 2014) and have become standard payment mechanisms (Fabian 2016). They are used globally in a society that views its transactions between direct parties and perceives them as being more straightforward and negotiable because monetary conversion is not needed (Kristoufek 2013).

### Privacy and security

Originally, virtual currencies emerged as a means of digital exchange that guaranteed their security, integrity, and balance due to a higher level of protection created by users. In exchange for compensation, these individuals help with security work by processing algorithms (Van Alstyne 2014; Urquhart 2018). That is, the security mechanisms of this payment method arise from the users themselves who maintain and protect the base fabric by providing computing power (Böhme et al. 2015). Mathematically speaking, the security of an electronic currency or the blockchain can be compromised, but the cost required to achieve this would be high, depending on the algorithm and its creation protocol (Xu 2016; Khan and Salah 2018; Zhang et al. 2019).

Transactions carried out with these currencies are direct between users and generally anonymous (Miers et al. 2013), compared with those carried out with legal currency in which payments are made through banking networks. Therefore, anonymity has been a key factor since their very inception (Ober et al. 2013). Although the development of cryptocurrency has not always been equal and not all types of cryptocurrencies operate the same, the complexity of violating anonymity is equal to the breach of their security (Wang et al. 2018). Privacy and protection are mechanisms that, although considered strong, need to be improved to add new functionality as they progress in their use because their standardization makes them attractive to hackers (Conti et al. 2018; Feng et al. 2019).

### Blockchain setup and maintenance

Electronic currencies are created through mining, an incentive process in which transactions are verified and new units are created and added to the core of existing ones (Eyal and Sirer 2013). The miners are responsible for collecting the latest transactions into blocks and finding a solution to the algorithm of each currency. As a reward, a fixed amount of that currency is acquired by these miners (Böhme et al. 2015; Bonneah et al. 2015). The solution to the algorithm changes continually and depends on previous results to perform the next calculation in the sequence. This means that, as time goes by, the difficulty in finding a solution will become greater, and its cost increases (Eyal and Sirer 2013; Giungato et al. 2017). Thus, the process has been affected because the investment cost does not exceed the profits offered (Kristoufek 2015; Cocco and Marchesi 2016).

All the information related to the cryptocurrency is recorded on the blockchain, a digital book shared on the network and responsible for collecting all the transactions carried out with the cryptocurrency in two parts (i.e., input and output) (Franco 2014). These exchanges or transactions are called blocks and are encoded and linked with others (Böhme et al. 2015). Blockchain information is stored on participating devices and is open access (Zyskind et al. 2015), making the exchange process transparent and immune to modifications (unalterable) (Brandvold et al. 2015). Once the data are verified, they can no longer be edited without the community’s consent. This recent technology in cryptocurrencies can be used for multiple purposes (Sikorski et al. 2017; Kuo et al. 2017; Lee 2017) and is one of the most dynamic elements of the economy (Yin et al. 2017).

### Challenges

Due to the simplicity of use (Selgin 2013) and the lack of regulation, particularly concerning taxation (Follador 2017), virtual currencies have been linked to numerous unregulated activities, including criminal acts, and may contribute to further price distortion (Barratt et al. 2013; Hardy and Norgaard 2016; Foley et al. 2019; Griffin and Shams 2020). Another problem with these currencies is their high level of volatility, losses, and a lack of widespread acceptance among the general public, which could indicate their inefficiency (Nadarajah and Chu 2017; Klein et al. 2018). Although volatility can mean both a risk and an opportunity (Brière et al. 2013), it is an intrinsic part of the currency (Bariviera 2017) and virtually impossible to predict (Balcilar et al. 2017). Recent studies have found that short-term bubbles limit the ability to profit from these tools; however, investments in these currencies are not limited, leaving only conjectures about obtaining economic benefits (Li et al. 2018). The continuous variations and collapse in the exchange of distributed volume generate large fluctuations in prices (Navas-Navarro 2015; Polaski et al. 2015) that denote the inefficiency of this market (Urquhart 2016; Zhang et al. 2018; Neslihanoglu 2021). It is an exchange mechanism whose real value starts from zero (Van Alstyne 2014; Cheah and Fry 2015). Although their permanence is currently being discussed as a matter of general interest, research has posited that the life cycle of cryptocurrencies increases, as they stabilize (Bariviera et al. 2017).

### The market and the protocols

Many virtual currencies have currently been given a relative value, based on different variables, to the different legal tender currencies (Table 2). All belong to a version of the protocol, depending on their application. Thus, we find that Bitcoin uses version 1.0 of the blockchain, whereas other alternatives, such as Ethereum, use version 2.0. The latest version, called version 3.0, is part of an extension of the applications used. Bitcoin and Ethereum have been chosen as the most relevant currencies based on their original protocols, which share several characteristics, such as mining or their structure; however, differences also exist between them (Table 3).

**Table 2:** Digital Currency by market value (July 2021)

Position	Name	Market cap	Price	Shares in circulation
1	Bitcoin	$750,600,171,509	$39,959.72	18,770,200 BTC
2	Ethereum	$271,733,293,394	$2,325.39	116,889,042 ETH
3	Tether	$61,828,690,396	$1.00	61,796,971,748 USDT
4	Binance Coin	$52,857,378,310	$314.44	168,137,036 BNB
5	Cardano	$41,218,509,234	$1.29	32,065,792,346 ADA
6	XRP	$32,843,217,941	$0.7071	46,312,443,360 XRP
7	USD Coin	$27,363,663,734	$1.00	27,354,066,325 USDC
8	Dogecoin	$26,668,380,056	$0.2044	130,639,341,482 DOGE
9	Polkadot	$14,779,410,550	$15.10	979,197,585 DOT
10	Binance USD	$12,228,250,268	$1.00	12,224,571,047 BUSD

*Source*: Own compilation. Data collected from CoinMarketCap

**Table 3:** Comparison between Bitcoin and Ethereum

	Bitcoin	Ethereum
Concept	Bitcoin is both a currency and a digital payment system	The Ethereum network is based on distributed ledger technology (DLT) or blockchain
Launch Date	31st of October 2008, date of publication of White Paper	December 2013
Form	Cryptocurrency	Cryptocurrency
Base	Blockchain	Blockchain
Ticker	bitcoin (BTC)	ether (ETH)
Purpose	Payment System	Allows execution of Smart contracts Contracts and decentralized applications by means of writing lines of code
Design	Virtual Currency	Token
Supply	Mining Recompense is based on validation of blocks	Mining Validation of blocks, transactions or contracts
In circulation	21 million bitcoin in total	18 million per year
Other	Used like any other fiat currency	Includes supplementary fees for “gas” Only works within its own network

*Source*: Own compilation

Bitcoin is the pioneering platform of the blockchain concept based on a peer-to-peer exchange that does not rely on traditional transaction schemes in which central authorities or banks carry out transactions. Bitcoin can be defined as a form of cryptocurrency or payment system based on cryptographic evidence whose unit is bitcoin (Nakamoto 2008) and has unique characteristics that have defined the properties of these currencies (Phillip et al. 2018). Having evolved from the Blockchain 1.0 protocol, Bitcoin is currently the most valuable and central axis of cryptocurrency studies (Jang and Lee 2018). However, it has shared its weight with those of recent creation.

Meanwhile, Ethereum is an open-source, decentralized platforms whose purpose is to create the most significant smart contract agreements (Luu et al. 2016). It is a framework for the execution of contracts and useful automated computer applications (Bhargavan et al. 2016), without the need to trust third parties. It is currently considered one of the most complex networks under review. We have chosen to analyze Ethereum in this study because it is one of the pioneering and most stable cryptocurrencies 2.0.

## Research methodology

The bibliometric analysis is responsible for reviewing different bibliographic material to organize the relevant information on a specific topic. It is also a way of presenting scientific publications that seek to assess the status of a given topic and the quality and influence of authors and sources (Van Raan 2014).

For the elaboration of the present study, we have followed a series of systematic stages. First, we established a list of research questions oriented for this study, which helped delimit the most important words, the search pages, and the chosen period, marking the direction of the work. Once the main theme had been structured and created, the first results were filtered, delimiting the research toward a total number of 1455 scientific articles distributed among the WoS and Scopus databases. With the obtained metadata, we then proceeded creating our own database which has been used for the present analysis.

## Research questions

We formulate research questions that can help us identify the volume of articles to predict future patterns and determine future lines of work to focus on. These questionnaires will also make us easier to determine which papers and publication venues to publicize our research. Lastly, these questions will help establish the relevance of the field at a general level and help find possible new funding or coordinated research avenues among the agents involved. We thus present the following research questions:

*Q1*: What is the distribution of publications on cryptocurrencies, especially Bitcoin and Ethereum, in relation to their citations?

*Q2*: What areas of publications have the highest impact?

*Q3*: Which articles are the most influential in this technology according to the number of citations, and where are they located?

*Q4*: Which are the most relevant and related countries and institutions?

### Data extraction

This study analyzed cryptocurrency, and the sources are the WoS and Scopus databases that include the largest number of academic journals and publications. It also analyzes the most frequently published authors, the most common or relevant topics, the number of publications by country, and the language used for the largest number of publications.

Two noteworthy sources have been chosen to solidify their documentary strength (Manterola et al. 2005). This study’s validity study depends on whether the subject area or the topic being researched is included in the sources of information. For many years, WoS was the only database designed as an international and multidisciplinary tool. Subsequently, Scopus was developed to compensate for the limitations of its predecessor, and to date, it is a more extensive database.

Based on several assumptions, the analysis is structured as follows: First, the parameters of the study were chosen or defined to select the appropriate databases from which to extract the data. Second, the corresponding search criteria were adjusted, and the bibliographic information categories were compiled. Finally, the extracted material was coded and used to create a combined database, and the extracted data were analyzed and contrasted.

**Fig. 1:**
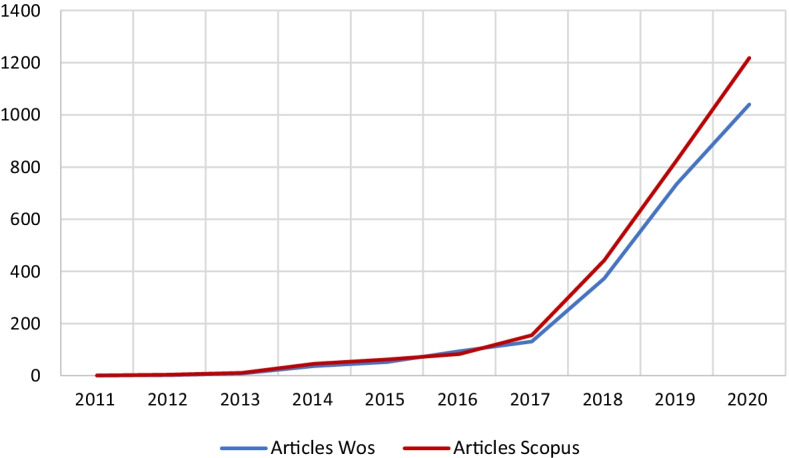
Annual scientific production. *Source*: Own compilation

The words selected for the search were “cryptocurrency,” “Bitcoin,” or “Ethereum.” This selection covered both the generic concept of electronic currency and the two types of pioneering and best-known currencies in the protocol’s respective version. The period selected was from 2010, the date of the first publication, to 2018, using the years 2019–2021 to check whether the published articles influenced future research trends. This is because, looking at all the data, we determine a turning point at which publications begin to double the number of the previous year (Fig. 1). From the aforementioned search criteria, we selected the filter for scientific articles as these were considered to be the most representative.

### Documents selection and data analysis

We have used three different indicators for the selection of documents: quantity, quality, and the structural form and the relationship between publications. Quantity shows the productivity index in terms of the number of publications. Meanwhile, quality shows which publications have the greatest impact according to the total number of citations received by a given text. Of the three, the two central ones of this text will be quantity and quality. These lead to the development and identification of successive rankings that will be displayed in various tables.

After selecting the documents to be used, we created three databases, that is, an individual one for each platform for comparison and a common unified one for specific analyses. For this, we have used three software packages: Tableau, R (Bibliometrix R Package), and VOSviewer.

The coding process was conducted by building a database using different variables that store information about each article, thereby extracting the productivity related to this research field.

Finally, after selecting the questions and extracting and preparing the data, we conducted an analysis consisting of the number of publications and their incidence, a selection of research areas, a distribution by country, institutions and journals, a more detailed section dedicated to their authors, and a summary of the trends.

## Results

**Fig. 2:**
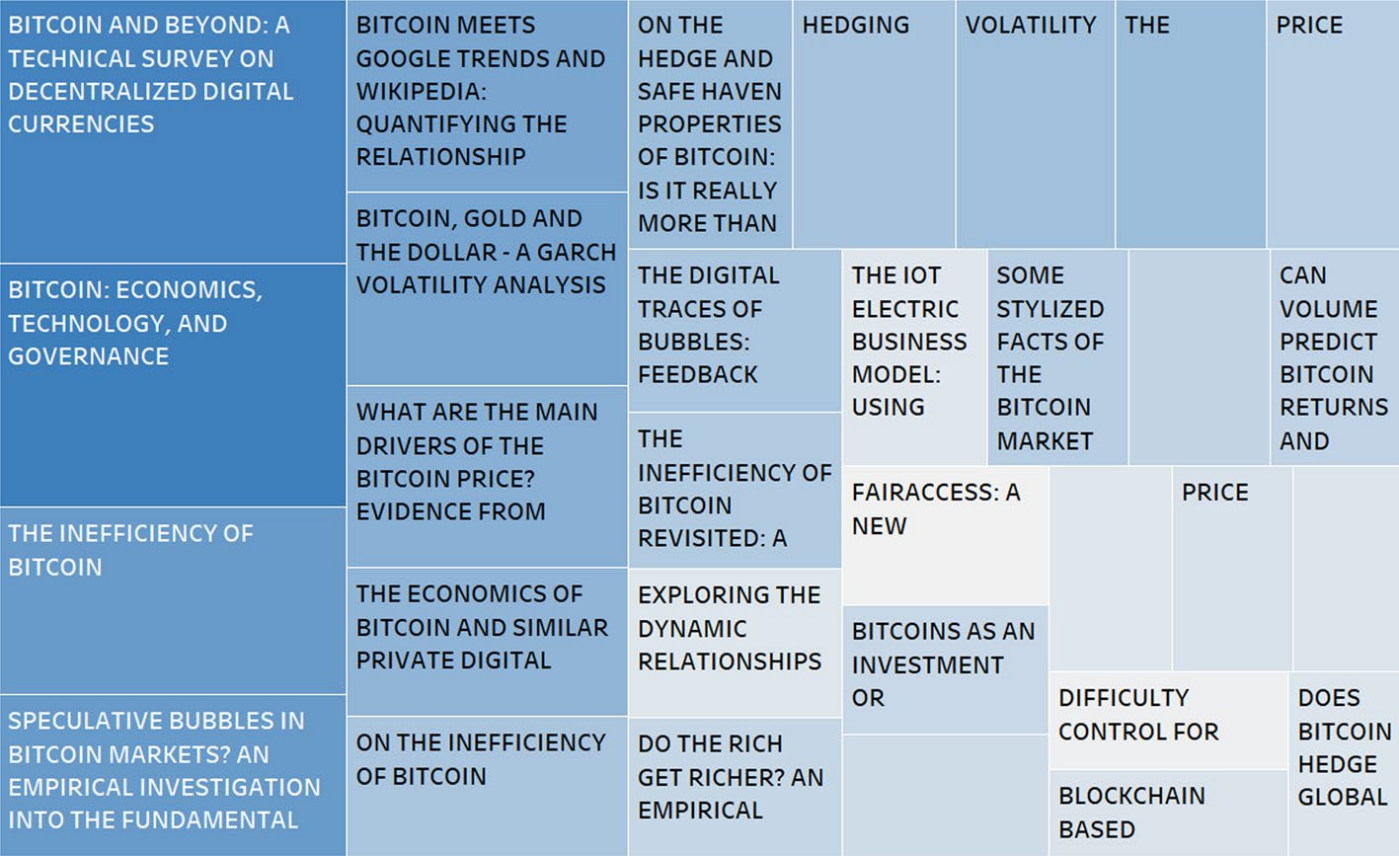
Most Cited articles in both databases. *Source*: Own compilation

To achieve a global view of the productivity in this field of research, this study’s results encompass the articles published during a given period and include information about their respective languages, countries, institutions, journals, and authors. As we have mentioned, the WoS and Scopus database search applies from 2010 to the end of 2018 because from 2019 onwards, the number of publications has multiplied, especially those related to the term blockchain, which may mislead the results (Fig. 2).

### Initial approach

The following data show the evolutionary state of the cryptocurrencies up to the present. As mentioned, the referenced sources are the WoS and Scopus databases, in which WoS is considered the pivotal source because of its greater seniority.

The first section analyzes the sample. Applying the corresponding search filters, we found 684 documents on the WoS database and 771 items on Scopus. Of these combined results, 407 documents appeared in both databases. The search in the two databases utilized the same period and began receiving content relevant to this study at almost the same time. Although the search is delimited by years, we focus on the starting year 2010 because of an anomalous result in Scopus in 1952 that coined the term Ethereum in an investigation by Dr. H. Greiner in the area of medicine. After excluding this search result, both bases coincide in the date of publication of articles, thus establishing this criterion equally.

Publications that included keywords, such as “Bitcoin,” “Ethereum,” or “Cryptocurrency,” appeared in 2011. Thereafter, the number of publications that included these keywords doubled annually. The recent creation of the aforementioned cryptocurrencies and their low impact indicate no related publications during the first years. Since 2011, when a single publication appeared in both databases, the results have increased exponentially. Figure 2 highlights that the trajectory followed by both databases is similar in terms of total publications, although with internal differences. If the set of publications is analyzed, Scopus includes a larger number than WoS, except for 2016, in which this trend is reversed.

The first publication included in WoS is “On Bitcoin and Red Balloons” (Babaioff et al. 2012), which talks about getting a reward in a node “competition.” Meanwhile, on Scopus, the first article is “Bitcoin: A bit too far?” (Jacobs 2011), which deals with issues internal to the currency. Although both publications received a low number of citations, the article “Bitcoin: A bit too far?” obtained a total of 10 citations compared to the two citations received by the article on WoS.

In terms of citations on both platforms, the most significant articles practically coincide, making it more relevant even with the creation of a common database that combines both sources (Fig. 2). In a separate analysis, both databases would show concordance in two of the three articles. Moreover, both articles would be in WoS and Scopus, although in different ranking positions. The article “Bitcoin: Economics, Technology, and Governance” (Böhme et al. 2015) is ranked first in WoS with 139 citations, whereas in Scopus, it is ranked third with a total of 207 citations. The article that ranked second on WoS is “Bitcoin and Beyond: A Technical Survey on Decentralized Digital Currencies” (Tschorsch and Scheuermann 2016), with 133 citations; however, this article is ranked first in Scopus, with a total of 225 citations. Meanwhile, the article “Where is current research on Blockchain technology?—A systematic review” (Yli-Huumo et al. 2016) ranks second on Scopus, with a total of 210 mentions, but it did not have any citations on WoS. Finally, the third-ranked article on WoS, that is, “Speculative bubbles in Bitcoin markets? An empirical investigation into the fundamental value of Bitcoin” (Cheah and Fry 2015), has 109 citations.

**Fig. 3:**
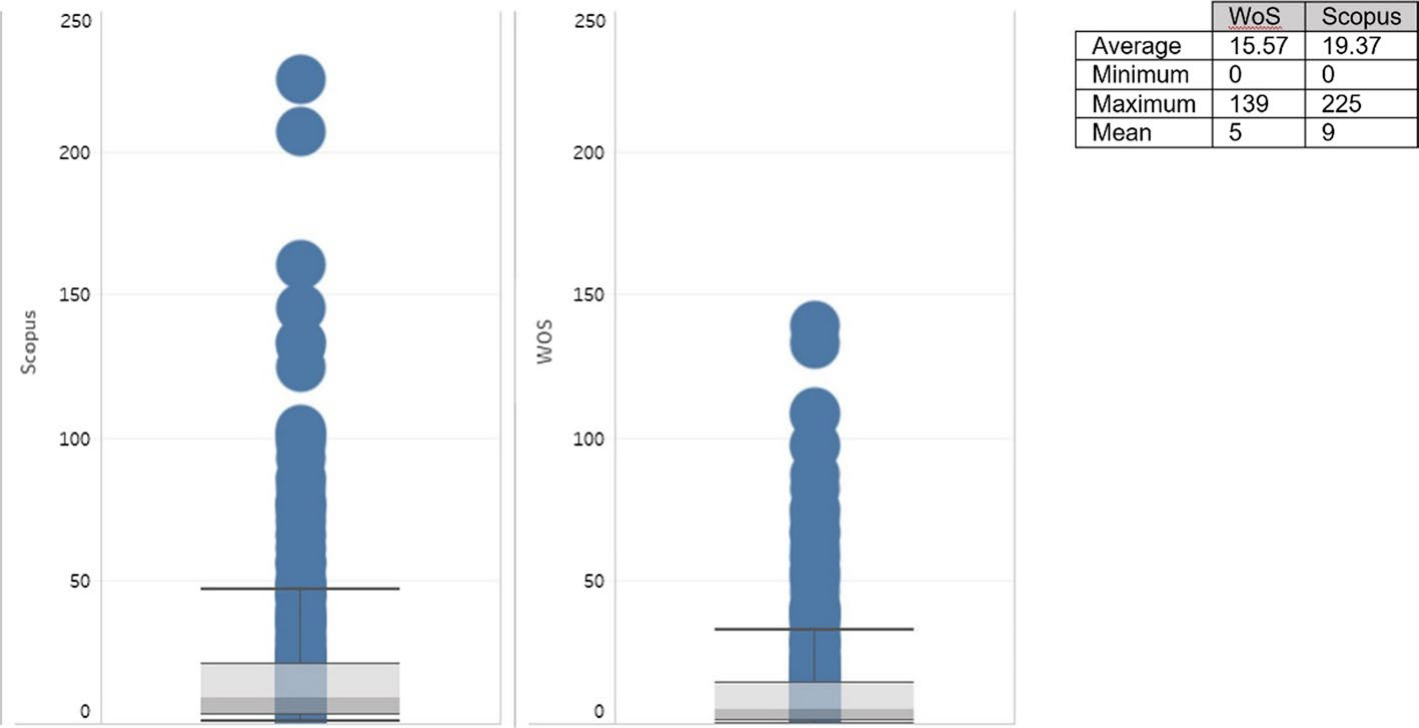
Comparison of citations. *Source*: Own compilation

Apart from the articles ranked first, the number of citations on Scopus is higher than on WoS (Fig. 3). The average number of citations per article is also higher, that is, 19 on Scopus compared to 15 on WoS, even though WoS contains a larger number of documents on the topic. Although both databases commence with articles without citations, the ends of the diagram show a greater number of atypical results in Scopus.

As can be seen, the results are quite similar, both being in an equal position. The country variable in both also shows a homogeneous growth and with similar results. The most significant distinction can be found in the total number of citations if the results are distributed over the years with a significantly higher number of citations on WoS. This is because although the number of articles is lower, the variables of authors and journals are higher (Table 4).

**Table 4:** General view

Year	A		Au		C		J		TC	
	*WoS*	*Sco*	*WoS*	*Sco*	*WoS*	*Sco*	*WoS*	*Sco*	*WoS*	*Sco*
2011	1	1	4	1	1	1	1	1	2	9
2012	2	4	2	4	1	1	2	4	32	52
2013	8	10	9	18	9	6	8	9	253	296
2014	37	44	33	76	14	21	21	36	245	489
2015	52	62	97	117	22	25	48	54	863	782
2016	87	81	166	159	33	35	70	66	1102	1129
2017	132	148	288	159	37	39	101	105	1193	860
2018	365	421	892	160	65	69	212	159	1258	623

A = Articles, Au = Authors, C = Country, J = Journals, TC = Total cites. *Source*: Own compilation

### Distribution by area of research

When comparing the databases, our search results show that the main areas of knowledge are information technology and economics (Table 5). Although WoS had 100 fewer results when the same number of research areas were considered, the wide range of classified thematic areas contained within WoS is greater than the classification in Scopus, and thus the articles are distributed across a wider range of subjects.

**Table 5:** Distribution by research area

RW	A	RC	A
Economics	156	Computer Science	269
Business finance	125	Economics, Econometrics and Finance	217
Computer science information systems	96	Social Sciences	188
Law	62	Engineering	166
Engineering electrical electronic	43	Business, Management and Accounting	133
Telecommunications	43	Mathematics	70
Computer science theory methods	42	Materials Science	39
Computer science software engineering	40	Decision Sciences	33
Multidisciplinary sciences	38	Biochemistry, Genetics and Molecular Biology	31
Computer science interdisciplinary applications	28	Arts and Humanities	29
Computer science hardware architecture	24	Physics and Astronomy	28
Business	22	Multidisciplinary	20
Physics multidisciplinary	20	Agricultural and Biological Sciences	16
Management	14	Energy	16
Remaining areas	209	Remaining areas	68

RW = Research area WoS, RC = Research area Scopus, A = Articles. *Source*: Own compilation

Using WoS as a reference, we use areas of economic knowledge, such as economics and business finance, in the ranking. The total sum of these articles is 282, which is similar to the second category in Scopus, which encompasses Economics, Econometrics, and Finance. The remaining positions in the ranking are related to computer science, systems, and telecommunications, almost half of those included in the list. The remaining articles are distributed among multiple categories, that is, a total of 76 different research areas include the terms Bitcoin, Ethereum or Cryptocurrency, although only 14 of these are specifically listed in the table. In contrast, Scopus directly links computer-related articles and ranks them first. Next, the economic and social sciences are ranked second and third with the remaining articles being linked, to a greater extent, to computer sciences, such as engineering and mathematics; and the social sciences with business and management. Once the threshold of the eighth theme is crossed, a greater diversity of topics begins to be seen.

The results of both the databases and the many thematic areas denote the wide variety of applications that technologies derived from electronic currencies have. Although the keywords are based on economics, the standardized use of technologies born from cryptocurrencies, most notably digital ledgers or blockchain, means that the distribution of themes is very widespread. The blockchain shows a positive evolution in databases, such as WoS, with a total of 692 results solely in articles in a period of just three years. The term “blockchain” did not receive citations until 2015, the year in which its development really took off. Hence, its importance is evident when compared to the origins in Bitcoin, because it has managed to equal the same number of articles in half the number of years.

### Distribution by country

**Fig. 4:**
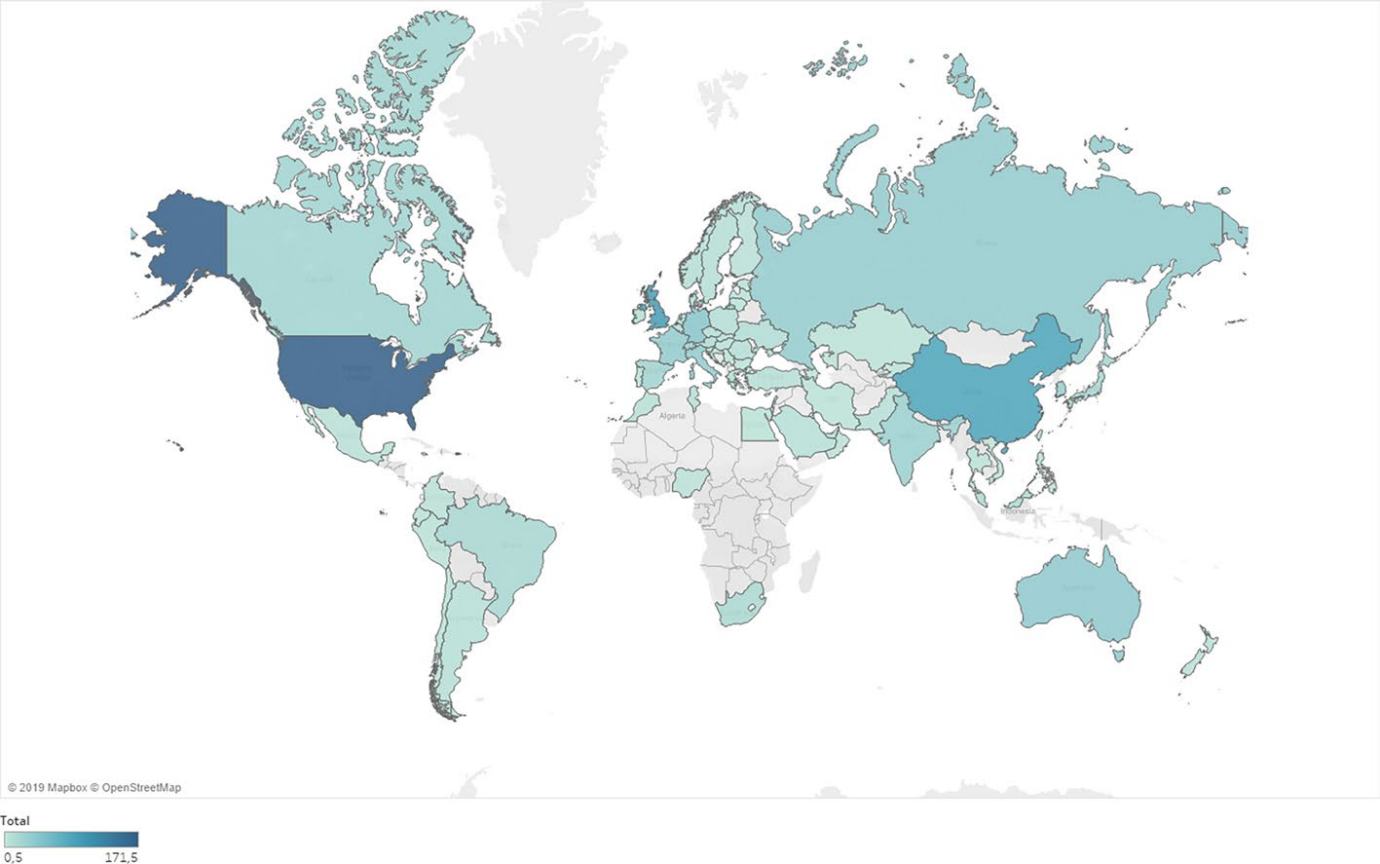
Geographical distribution. Mercator projection Map. Landmasses appear larger the farther they are from the poles. The projection, however, maintains constant bearings for navigation. *Source*: Own compilation

**Fig. 5:**
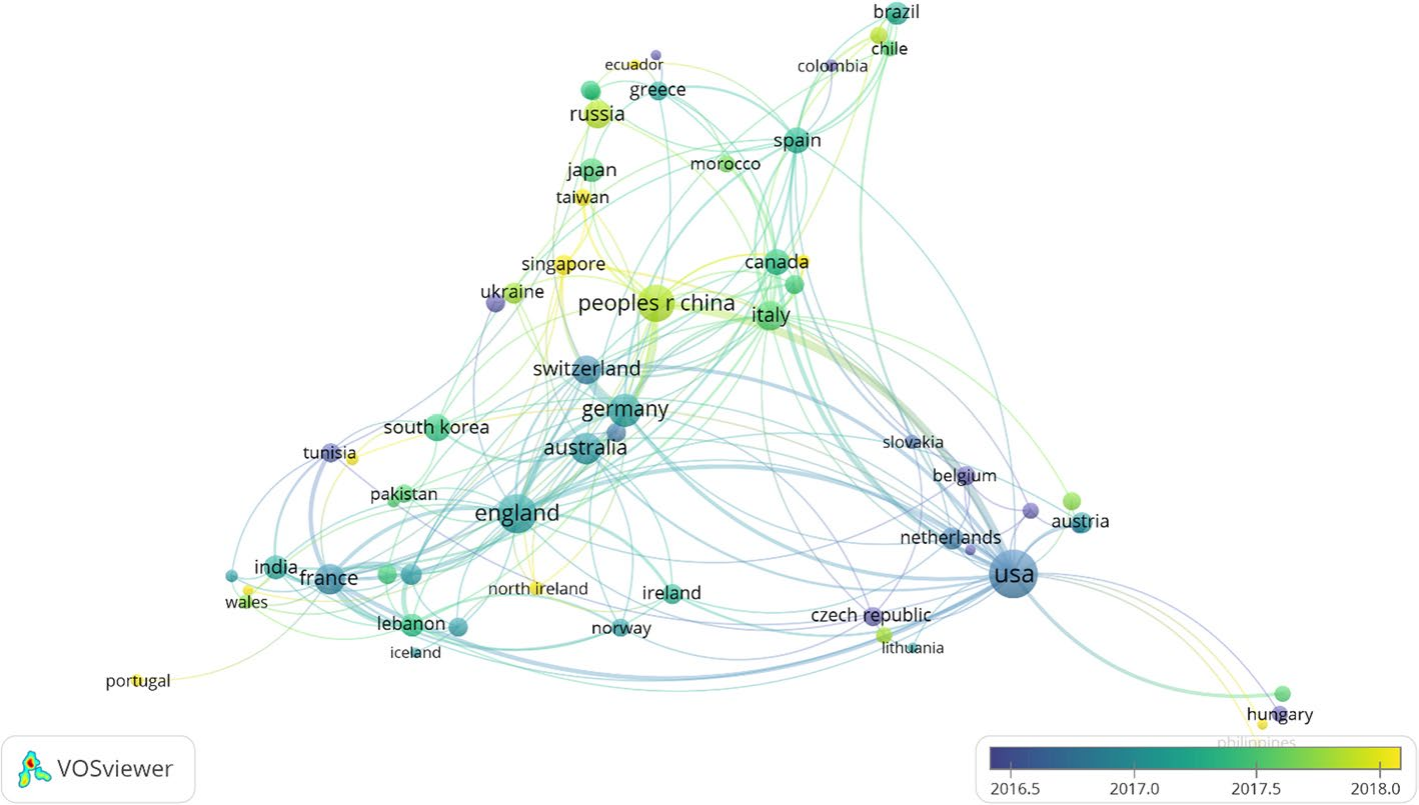
Grouping by country and year. *Source*: Own compilation

In terms of geographical distribution, an apparent growing trend toward research on this topic originates from the Asian continent, apart from the time factor (Figs. 4 and 5). That said, the principal language used is still English, and virtually all articles appear in the two databases published in this language. Other articles were published in Russian, Spanish, and Turkish in WoS, whereas the most used languages were Chinese, Russian, and German in Scopus. Note that although both databases consider Russian to an influential language, as a geographical region, Russia is not featured as one of the most influential countries in terms of the number of publications.

In a more detailed comparison, both databases show similar results with respect to the first four and the last two ranked countries (Table 6). Both databases show the USA, UK and China leading the ranking. These countries also account for the largest number of articles and citations together with the highest H-indexes. The databases also coincide with respect to the countries ranked last, with the possible exception of India, which in Scopus, is ranked sixth. Specifically, considering the ranking in terms of the number of articles published, the results from both databases practically coincide, whereas the results are more disparate in terms of the total number of citations. The discrepancy mentioned earlier in India can only be highlighted in the number of articles. Regarding the total number of citations, the rankings of Russia and Spain stand out for different reasons. In the case of Russia, the total number of citations is much lower than expected given the number of articles published. In contrast, Spain obtained a number of citations that would place it in several higher positions compared to the number of published articles; the h-index is clearly higher than that obtained in the classification.

**Table 6:** Distribution by country

C	R		A		TC		H	
	WoS	Sco	WoS	Sco	WoS	Sco	WoS	Sco
USA	1	1	182	161	1249	865	17	21
UK	2	3	84	91	1035	806	16	23
China	3	2	74	97	358	320	9	13
Germany	4	4	43	38	416	399	10	13
Australia	5	8	39	31	361	312	12	14
France	6	10	36	29	425	227	12	12
Italy	7	7	32	33	210	128	8	9
Switzerland	8	11	29	23	217	190	8	8
Russia	9	5	25	38	27	65	2	6
South Korea	10	9	23	31	136	175	7	8
Canada	11	12	22	19	70	79	5	8
Spain	12	13	22	19	350	235	8	10
India	13	6	17	33	105	63	5	7
Japan	14	15	15	16	51	42	4	5
Brazil	15	14	13	16	21	29	3	3

C = Country, R = Position in the Ranking, A = Articles, TC = Total cites, H = H-Index. *Source*: Own compilation

If we develop the content dealt with in each country in a more important way, taking a total of 5 words as the focus of studies, we can see how the USA has always studied bitcoin, deriving from it the concept of currency, blockchain, innovation, and economy together with security. For its part, and also taking bitcoin as a central focus, England has added the volatility of these currencies together with their technology, such as blockchain, to its most relevant words. China is next, giving the same importance to bitcoin as to the blockchain, deriving two lines of research from which the main concern of bitcoin comes from its inefficiency and prices; however, the blockchain mentions security and smart contracts. Germany and Australia are next on the list, but the main focus is on bitcoin, but it is much shorter in terms of secondary issues, just mentioning economics and blockchain. Meanwhile, Russia remains with bitcoin and cryptocurrencies in general and, if the number of keywords is lowered as a concurrence, China appears as another result, being the only ones to mention another place directly.

### Institutions

The most pivotal institution related to electronic currencies that focuses on Bitcoin and Ethereum is the University of London with a total of 24 and 14 articles in WoS and Scopus, respectively (Table 7). This institution is followed by PDX Currency Corp in WoS, with 17 published articles, although no citations are related to them. Again, in terms of number of published articles, the next ranked institutions are the University College London with 14 articles and Eidgenössische Technische Hochschule Zürich (ETH Zurich) and the University of California System with 13 articles each. They have also attracted a large number of citations. Except for ETH Zurich, the aforementioned institutions are all English-speaking, which coincides with the high number of publications in that language.

**Table 7:** Institutions

I	R		A		C	TC		AC		H	
	*WoS*	*Sco*	*WoS*	*Sco*		*WoS*	*Sco*	*WoS*	*Sco*	*WoS*	*Sco*
University of London	1	1	24	14	UK	162	73	6.75	5.21	7	6
PDX Currency Corp	2	–	17	–	USA	0	–	0	–	0	–
University College London	3	–	14	–	UK	74	–	5.29	–	4	–
ETH Zurich	4	4	13	9	Switzerland	147	158	11.31	17.6	4	5
University of California System	5	–	13	–	USA	105	–	8.08	–	4	–
Holy Spirit Univ Kaslik	–	5	–	9	Lebanon	–	117	–	13	–	8
Montpellier Business School	6	2	12	12	France	254	132	21.17	11	7	9
Beihang University	–	6	–	8	China	–	20	–	2.5	–	3
Languedoc Roussillon Universites Comue	7	–	11	–	France	251	–	22.82	–	7	–
University of Pretoria	–	7	–	8	South Africa	–	61	–	7.63	–	6
Chinese Academy of Sciences	8	3	10	11	China	49	158	4.9	14.36	5	6
Xidian University	–	8		8	China	–	17	–	2.13	–	3
University of Manchester	9	–	9	–	UK	117		13	–	4	–
Imperial College London	–	9	–	7	UK	–	28	–	4	–	4
Centre National de la Recherche Scientifique Cnrs	10	–	8	–	France	20	–	2.5	–	3	–
Ku Leuven	–	10	–	6	Belgium	–	63	–	10.5	–	4

*Source*: Own compilation

I = Institution, R = Position in the Ranking, A = Articles, C = Country, TC = Total cites, AC = Average citation, H = H-Index

In contrast, Scopus shows a greater spatial distribution with respect to institutions. Although the first result coincides with the aforementioned results from WoS, the institutions appearing next in the ranking are Montpellier Business School, Chinese Academy of Sciences, ETH Zurich and Holy Spirit Univ Kaslik located, respectively, in France, China, Switzerland and Lebanon. The articles published by these institutions have a higher number of references compared with more prominent institutions listed in WoS.

**Fig. 6:**
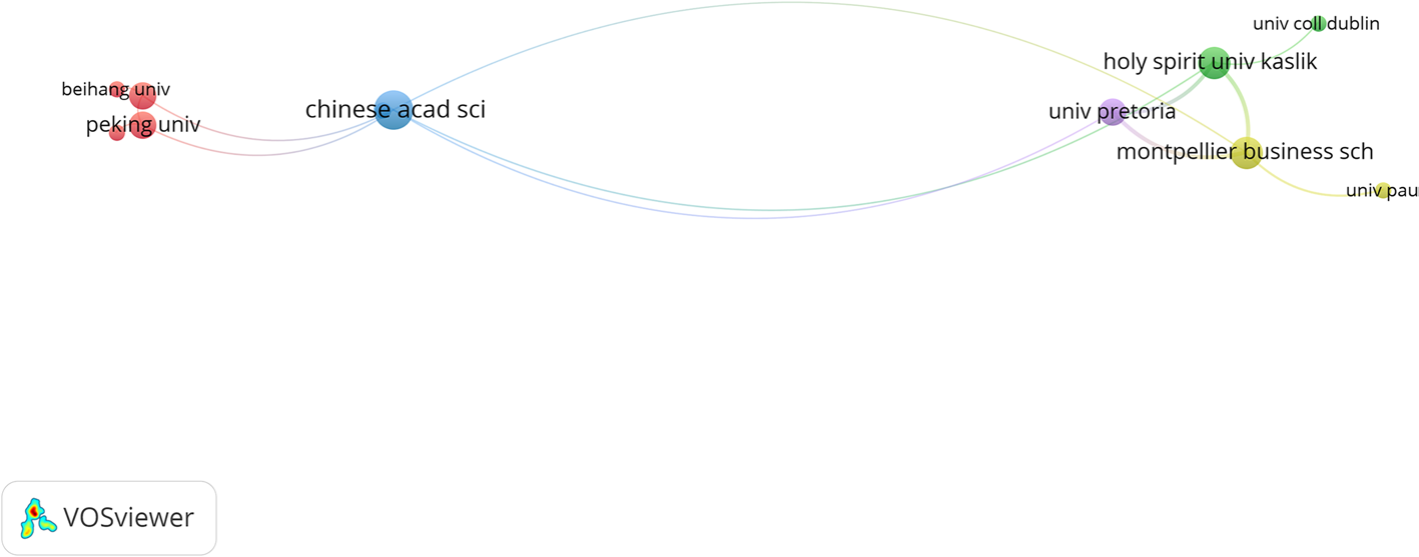
Cluster map of institutions. *Source*: Own compilation

The results show that WoS has a greater concentration of English and American institutions as a central pillar, bringing together a core of English-speaking institutions that makes up 40% of the total. In contrast, Scopus has a more varied distribution. The central focus of the five institutions of each essential database has always been on issues related to bitcoin as a core, with publications on its volatility, hedge, and economics deriving from it. In a more minor way this time, the concept of the blockchain appears. To conclude this section, we created a cluster map of institutions. As suggested by Fig. 6 and given the recent development of the topic, the links and relationships between institutions are scarce, with only a suggestion of a rapprochement between Asian entities.

### Journals

The journals with the highest number of publications in WoS and Scopus are Economics Letters and IEEE Access with a total of 29 and 28 publications, respectively in the case of Economic Letters and 26 and 30 in the case of IEEE Access. They both clearly have a high H-Index along with a large total number of accumulated citations. Two sources appear in the third position of the ranking of both databases, albeit without any associated citations. They are Digital Currency Challenge Shaping Online Payment Systems through US Financial Regulations and Economist United Kingdom with 17 and 21 articles, respectively from USA and UK. This phenomenon of not receiving any citations is repeated in the WoS ranking with the fourth ranked journal, Palgrave Pivot, and in Scopus with the seventh ranked journal, Technology Review.

In the sample provided, only five journals are considered global publications in Table 8 for both databases. This is evidence of the disparity between the two sources because, aside from the two journals mentioned in the previous paragraph, *Finance Research Letters, PLOS One, and Physica A: Statistical Mechanics and its Applications* are the only journals listed in both sources. Although there are no other concurrences, the basic scheme observed is remarkably similar because the coincident entities do so in almost an equal number of the ranking, whereas the remaining journals coincide approximately in the number of articles. The number of publications in these journals is always related to economics, inefficiency, volatility, and gold, leaving blockchain and security as secondary topics.

**Table 8:** Distribution by Journal

J	R		A		JCR	SJR	C	TC		AC		H	
	*WoS*	*Sco*	*WoS*	*Sco*	*WoS*	*Sco*		*WoS*	*Sco*	*WoS*	*Sco*	*WoS*	*Sco*
Economics Letters	1	2	29	28	0.876	0.767	Switzerland	721	377	24.86	13.46	13	16
IEEE Access	2	1	26	30	4.098	0.609	USA	111	63	4.27	2.1	7	10
Digital Currency Challenge Shaping Online Payment Systems through US Financial Regulations	3	–	17	–	–	–	USA	0	–	0	–	0	–
Economist United Kingdom	–	3	–	21	–	0.100	UK	–	0	–	0	–	0
Palgrave Pivot	4	–	17	–	–	–		0	–	0	–	0	–
Finance Research Letters	5	4	14	14	1.709	0.770	USA	405	242	28.93	17.29	9	11
PLOS One	6	5	14	14	2.776	1.100	USA	219	319	15.64	22.79	7	10
Physica A: Statistical Mechanics and Its Applications	7	6	13	13	2.5	0.699	Netherland	113	61	8.69	4.69	4	7
Technology Review	–	7	–	9	–	0.117	USA	–	0	–	0	–	0
ERCIM News	8		8					1		0.13		1	
Computer		8		8	–	0.498	USA		61		7.63		6
Journal of Risk and Financial Management	9	–	8	–	–			27		3.38		3	–
Royal Society Open Science	–	9	–	8	–	1.131	UK	–	25	–	3.13	–	2
Ledger	10	–	8					7		0.88		2	
Computer Fraud and Security	–	10	–	7		0.177		–	36		5.14		3

*Source*:  Own compilation

J = Journal, R = Position in the Ranking, A = Articles, JCR = Journal Citation Reports, SJR = Scimago Journal Rank, C = Country, TC = Total cites, AC = Average citation, H = H-Index

### Authors

As a final comparison, Table 9 shows the authors ordered according to the index of publications on the topic. The 17 articles by P.C. Mullan, which appear solely in WoS, can be highlighted as an anomalous result, as they have received no citations. This can be linked to the previous section on publications, as these articles are contained in a manual. Regarding the rest of authors, E. Bouri and D. Roubaud stand out with nine articles each, published in 2017 and 2018. Both authors have collaborated extensively and had many citations, well above the average of other authors, although not in all articles.

**Table 9:** Distribution by author

Au	R		A		TC		AC		H		FP	LP
	*WoS*	*Sco*	*WoS*	*Sco*	*WoS*	*Sco*	*WoS*	*Sco*	*WoS*	*Sco*		
Mullan, Pc	1	–	17	–	0	–	0	–	0	–	2014	2014
Bouri, E	2	1	9	9	223	117	24.78	13	7	8	2017	2018
Roubaud, D	3	2	9	9	242	131	26.89	14.56	7	8	2017	2018
Androulaki, E	4	–	7		29		4.14	–	1	–	2015	2016
Gupta, R	5	3	7	6	116	60	16.57	10	5	6	2017	2018
Luther, Wj	6	5	7	5	76	39	10.86	7.8	5	4	2016	2018
Wang, J	7	–	7	–	36	–	5.14	–	3	–	2018	2018
Bouoiyour, J	–	7	5	4	70	37	14	9.25	5	4	2015	2018
Karame, G	8	–	6		0		0		0		2016	2016
Corbet, S	–	8		4		27		6.75		3	2017	2018
Marchesi, M	9	4	6	6	41	22	6.83	3.67	4	4	2017	2018
Li, X	–	9	5	4	43	4	8.6	1	3	3	2017	2018
Selmi, R	10	6	6	5	71	37	11.83	7.4	5	4	2015	2018
Liu, J	–	10	–	4	–	4	–	1	–	2	2017	2018

*Source*: Own compilation

AU = Author, R = Position in the Ranking, A = Articles, TC = Total cites, AC = Average citation, H = H-Index, FP = First publication, LP = Last Publication

**Fig. 7:**
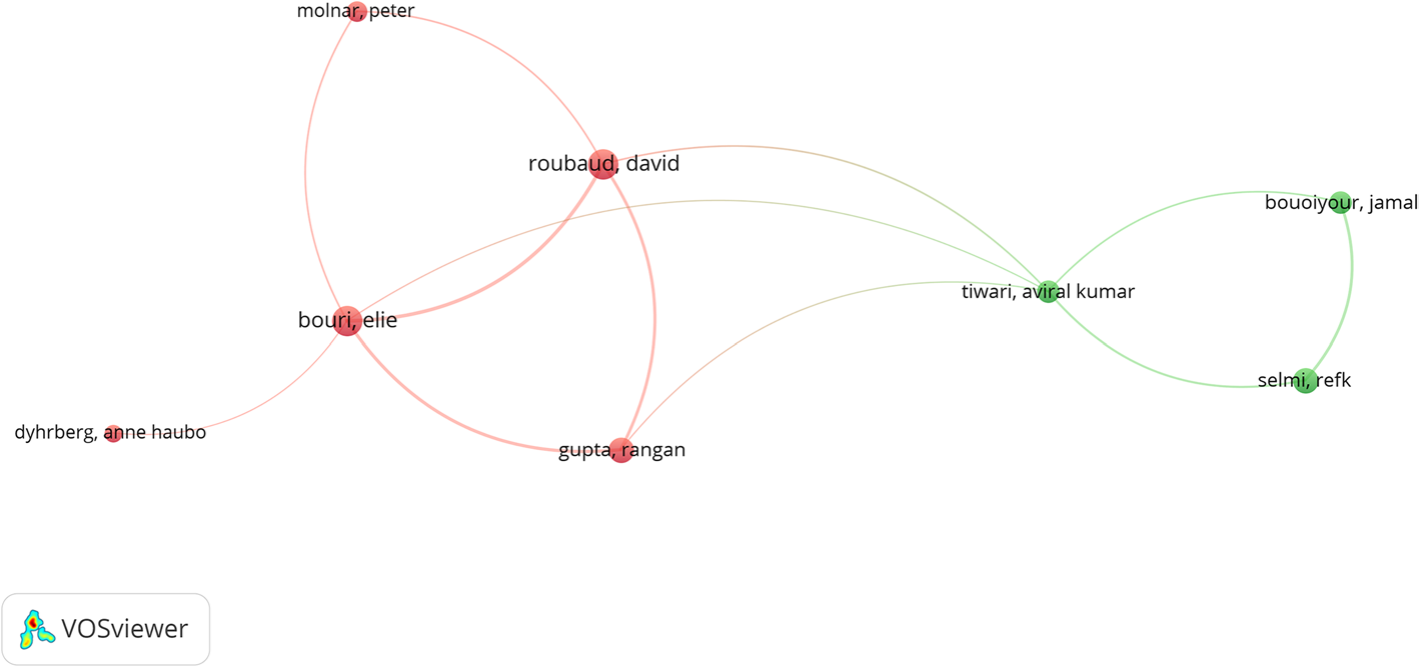
Author cluster on WoS. *Source*: Own compilation

**Fig. 8:**
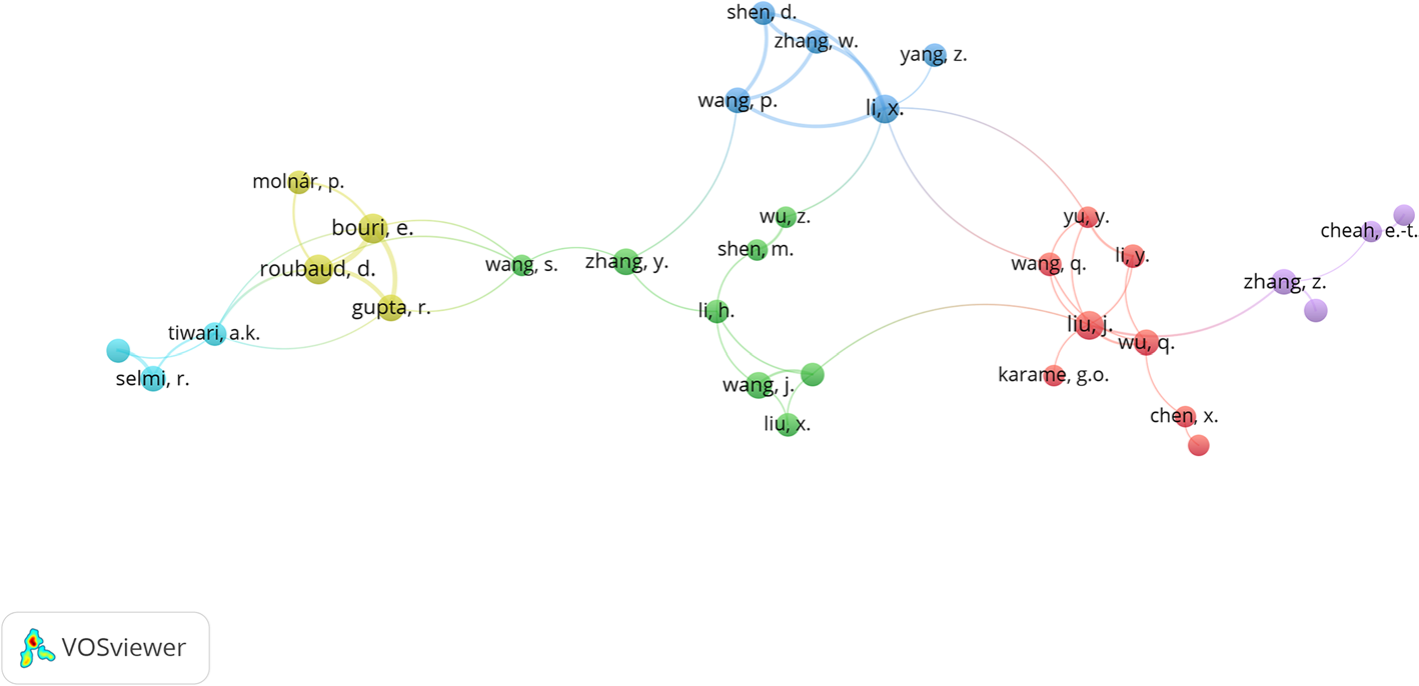
Author cluster on Scopus. *Source*: Own compilation

Based solely on the total number of publications, and focusing on the most influential authors, the distribution of authors in both databases is quite similar. Regarding the field to which the authors belong, the most important ones come from Business & Economics, Computer Science and Environmental Sciences & Ecology. However, in a cluster analysis (Figs. 7 and 8) and using the two databases as the basis for the analysis, we determine that the relationship between them changes. In both cases, the grouping has been generated using the same basic parameters that, together with the greater distribution among the Scopus institutions, shows broader results with six central nuclei versus the two mere nuclei in WoS.

**Fig. 9:**
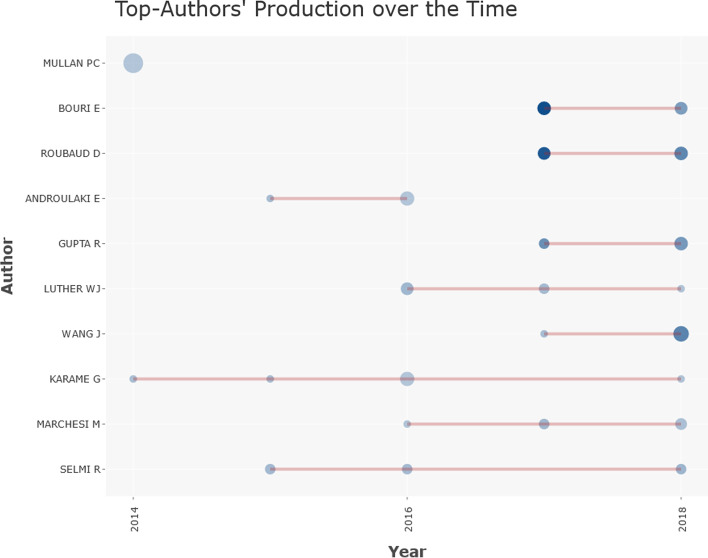
Top authors’ production. *Source*: Own compilation

Figure 9 shows the evolution of the scientific production achieved by the most relevant authors, taking WoS as a reference to observe their trajectory. The circles on the cluster map represent the number of articles, and the color represents the intensity of the citations received during the year. This would show how the most important publications were produced in WoS during 2017, coinciding precisely with the beginning of the increase in scientific publications.

### Trend analysis

**Fig. 10:**
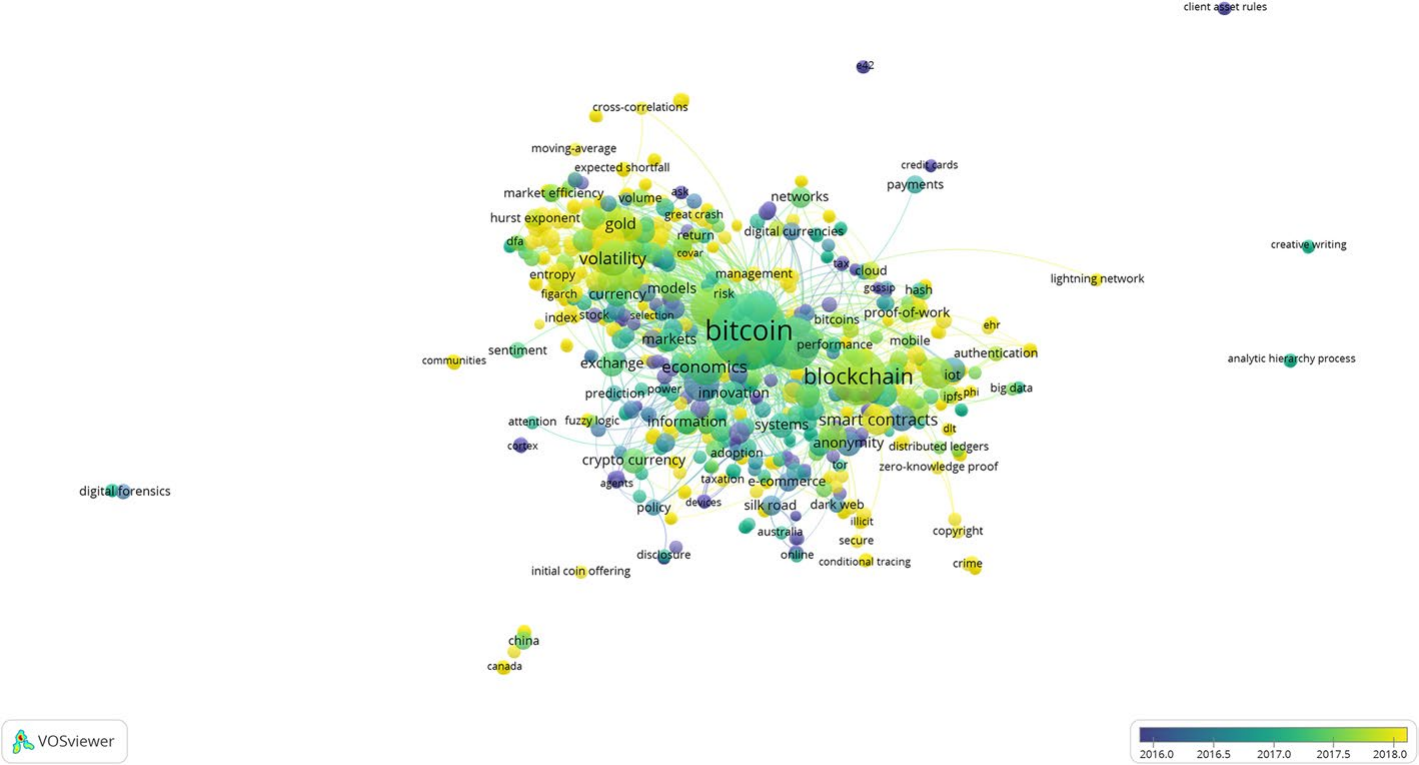
Keywords on WoS. *Source*: Own compilation

**Fig. 11:**
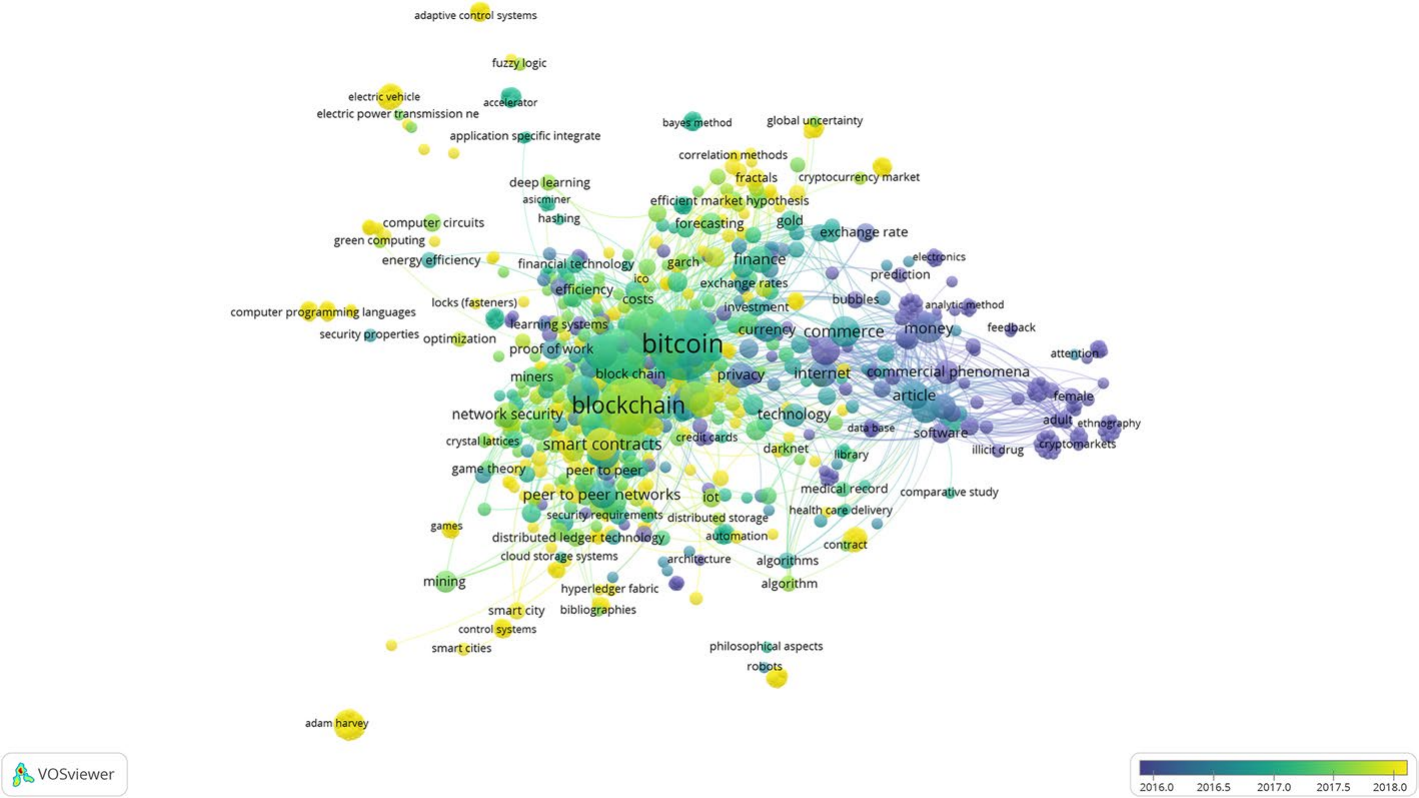
Keywords on Scopus. *Source*: Own compilation

**Fig. 12:**
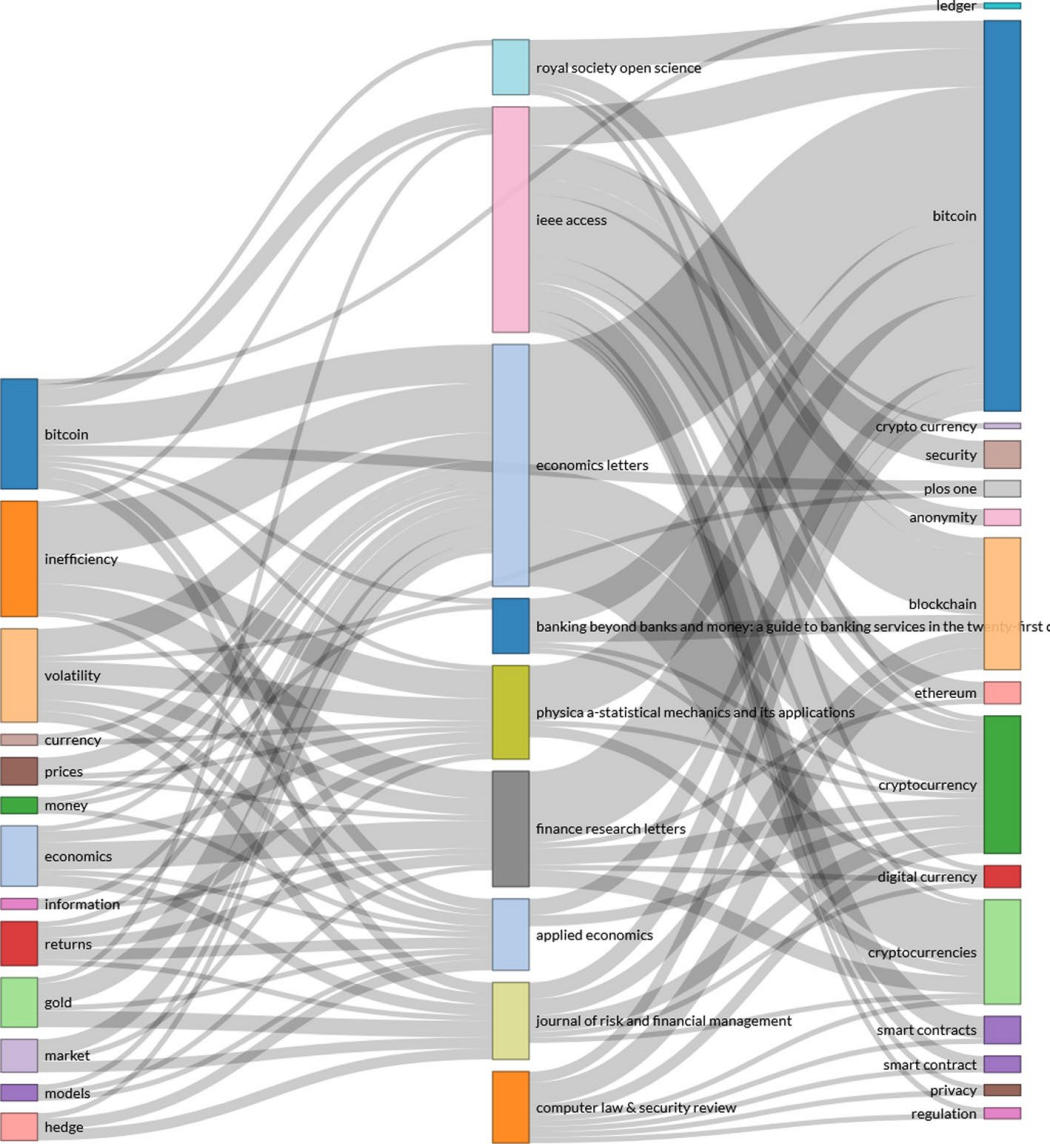
Three-fields plot of top Keywords plus, sources, and Author keywords. *Source*: Own compilation

Based on the content of all the articles, we can identify the most common terms and those with the greatest impact related to electronic currencies. Using the VOSviewer software and R (Bibliometrix package), we compiled a series of large clusters indicating the frequency and evolution of the keywords (Figs. 10 and 11), combined with a three-field plot of top Keywords Plus, Sources, and Author Keywords (Fig. 12). Notably, the wide variety of terms in Scopus is due to a higher index of publications, even if some of them have not been followed up.

The results of both graphs show similarities in terms of key concepts that are maintained over time. The secondary issues continue to have Bitcoin as the central focus, drifting toward the concepts of blockchain, money, and security. Remarkably, although the term Ethereum has been used as a study keyword, it does not appear directly in the cluster figures, although the derivative terms, such as Smart contracts, appear as the purpose of this type of currency.

The concept of security appears directly related to electronic currency, and hence, the fact that it is not reflected in any type of legal regulation is conspicuous, given the complexity of these payment mechanisms. If the latest publications and texts taken from conferences are incorporated, changes are made to the graph that had not been previously considered, such as security becoming an impactful mainstay of the topic. This is due to the standardization and greater acceptance of these types of currencies that had even been temporarily banned in countries, such as China (2019), which is now one of the largest producers of articles related to the subject, coming to appear in the keywords of both databases, although the current situation in China is complex, as its uses have recently been limited (China 2021).

Returning to the concept of security, we determine that the term crime appears close due to the increase in publications related to criminal acts, such as money laundering processes, darknet shops, or payment to ransomware, that in the last three years has doubled the number of publications (Turner et al. 2019; Albrecht et al. 2019). This terminology is related to the illicit and dark web keywords that evolve from the concept of anonymity.

To finish with the new trends section, we compiled a Sankey diagram (Fig. 12). The diagram shows the relationship between sources (center), Keywords Plus (left), and Author Keywords (right), which is especially useful for locating the topic in each of the journals (Riehmann et al. 2005). The size of the nodes represents the frequency of the item and the lines show the connections between them. The use of Keywords Plus and Authors' keywords shows a difference to be considered, as Keywords Plus are more effective than words given by authors in bibliometric analyses even if they are less representative of the article’s content. (Zhang et al. 2016).

We can argue that *Economics Letters* relates its publications to a greater number of terms, such as inefficiency, volatility, or market, covering more topics or characteristics because of connector flows. These are in turn closely related to the words “electronic currencies, bitcoin and smart contracts” as the authors’ keywords. Therefore, although this first node mentions more topics, they are all related to the economic world, leaving in the background the importance of applied technologies, such as blockchain. The publications of the second most influential node, *IEEE Access*, are closely related to the concepts of “inefficiency, Bitcoin, and volatility,” with special interest in the authors' words “bitcoin, security, blockchain, smart contracts, privacy and privacy regulation.” Therefore, the authors of these publications can focus more on the financial applications that arise from blockchain networks than on developing the currencies themselves. This perspective seems to be shared by four other sources (i.e., *Computer Law & Security Review, Journal of Risk and Financial Management, Banking Beyond Banks and Money, and Royal Society Open Science*), whereas *Applied Economics and Finance Research Letters* follow the trend of the first node.

## Discussion

**Fig. 13:**
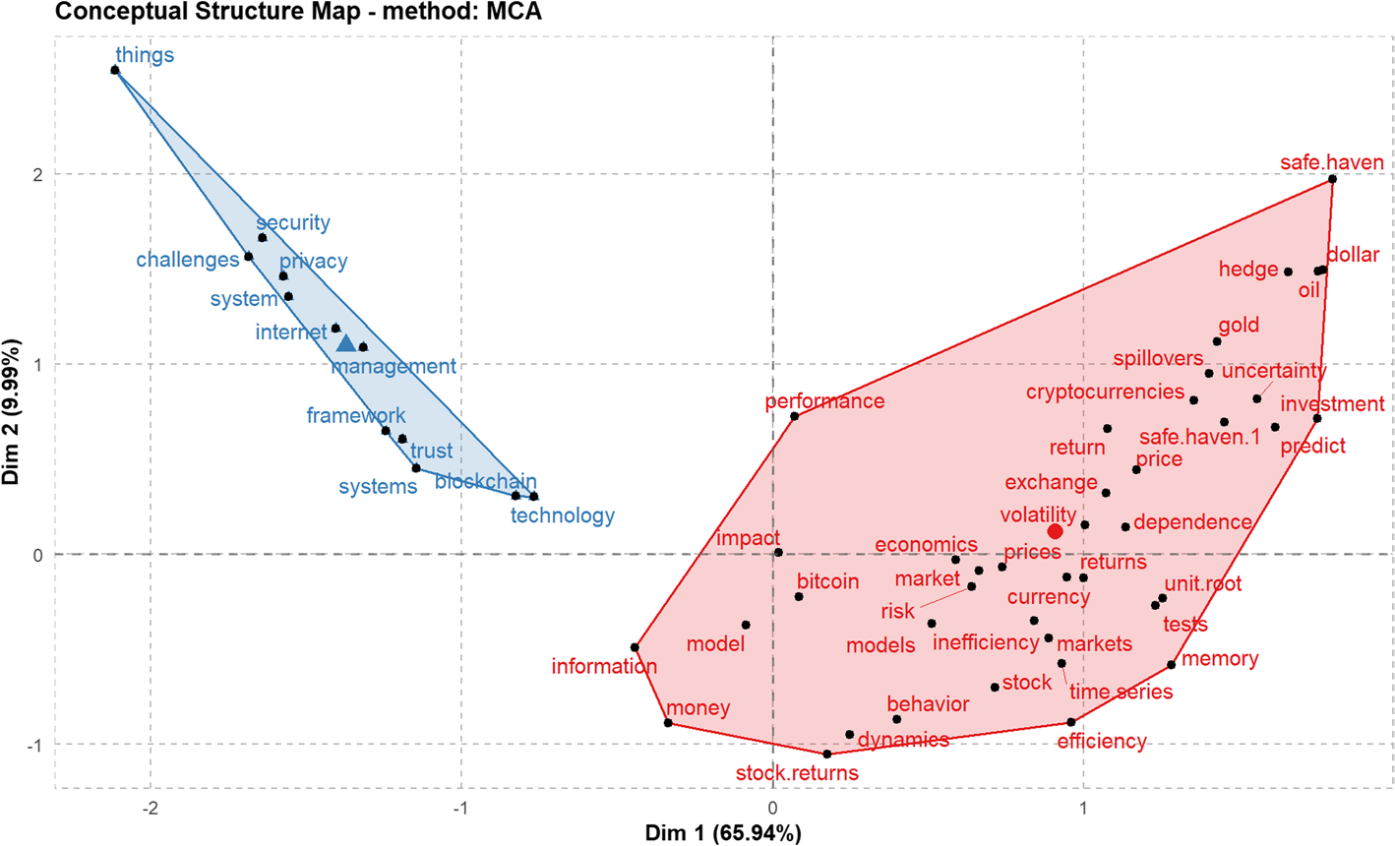
Conceptual structure map using MCA method. *Source*: Own compilation

**Fig. 14:**
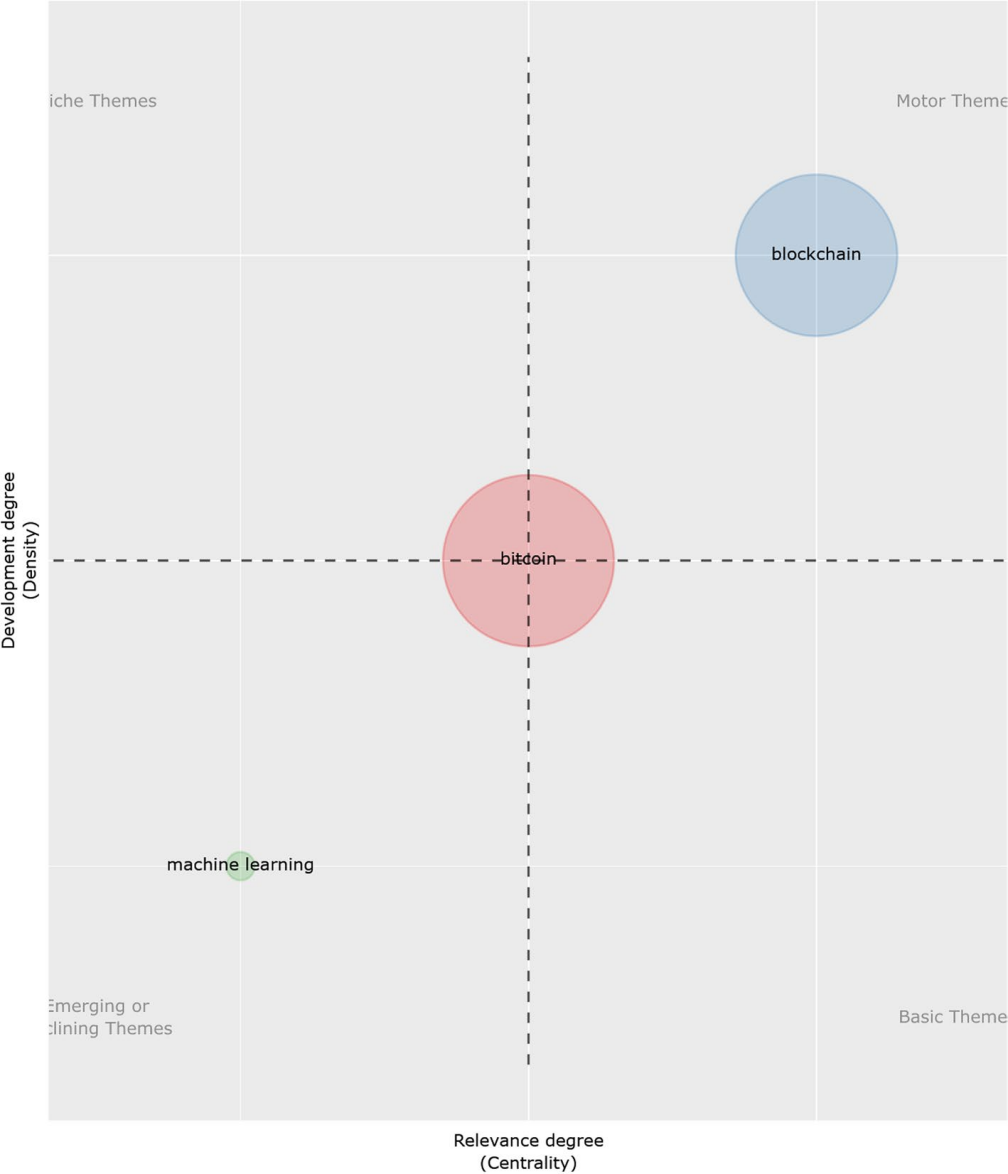
Thematic map using porter's derivation algorithm method. *Source*: Own compilation

Using 2020 as a deadline, we can see that the aforementioned trends are the ones that have finally concentrated on these publication types. The conceptual structure map (Fig. 13) of the MCA keyword plus method shows two main clusters in different colors that coincide with the driving themes of these publications (Fig. 14). This word clustering allows us to identify from today the groups with the same meaning and their relationships. Porter's derivation algorithm has been used to reduce the number of words used in a root form, but this time, from the authors’ keywords with similar results. In both cases, a maximum of 250 words per term has been applied. Both show that regardless of the analysis used and keywords, the central topics are Bitcoin and the blockchain network, which creates and supports the need for a separate bibliometric review of different areas to check the trends in them in the future. This situation is repeated in the different analyses conducted on the subject regardless of the basis used, clearly showing a separation between technology and economy (Merediz-Sola et al. 2019; Shen et al. 2020).

**Fig. 15:**
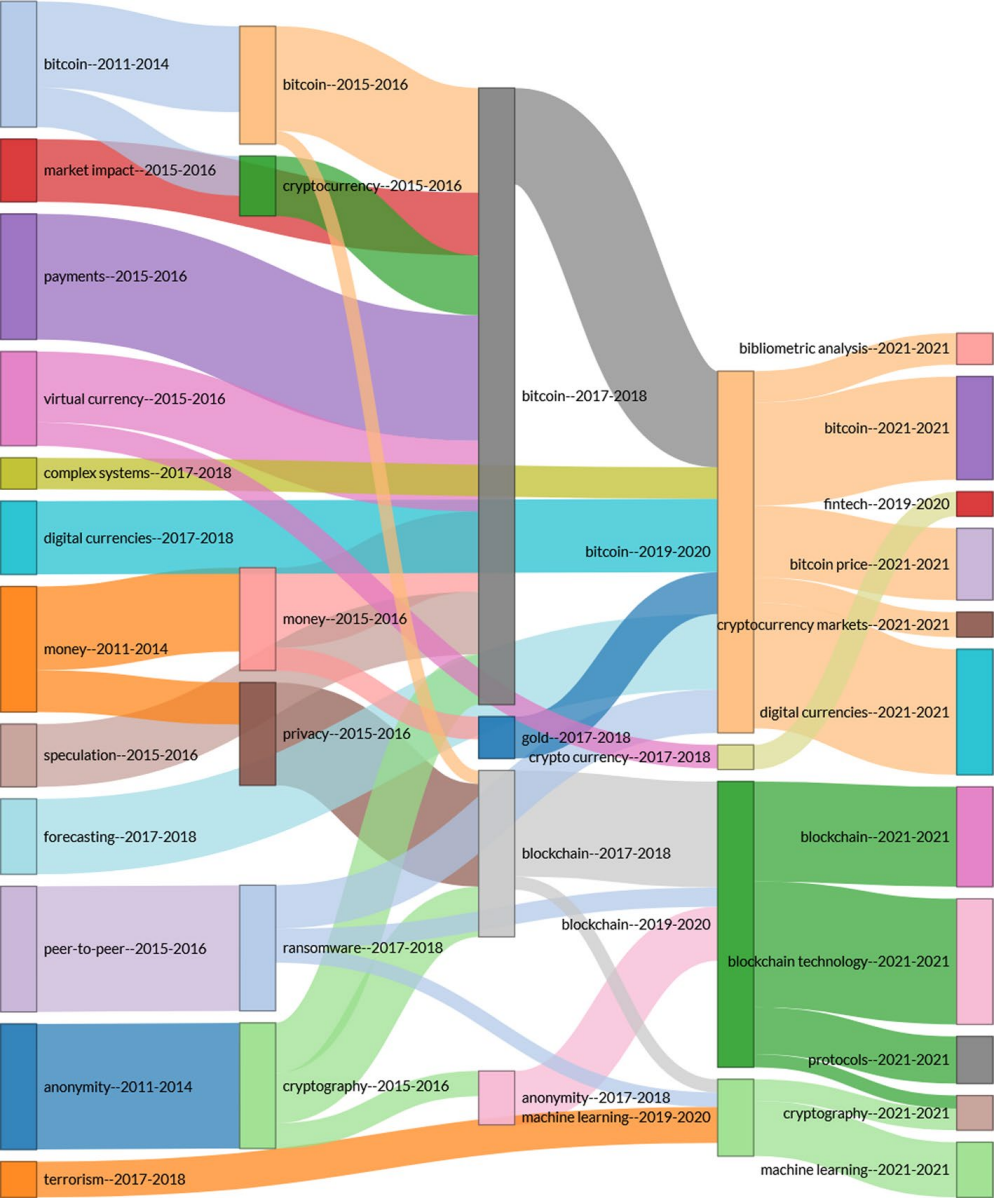
Topic evolution research (2010–2020). *Source*: Own compilation

Figure 15 shows a thematic division into four different periods. This is conducted to clarify how the same area has been clearly divided into two distinct interconnected branches since 2017–2018, creating the aforementioned economic-technological division. Although the concern for cryptocurrencies is related to their value in the market, technological evolution has opened up new lines of research thanks to its multiple applications, such as machine learning.

At this point, some questions arise:

What should be the way forward for cryptocurrency research?

Cryptocurrencies will continue to be published, following the concepts of volatility, decentralization, and efficiency as characteristics, along with the smart contract as an application that initiated the 2.0 protocol. Especially in this context, the concept of efficiency or inefficiency should be emphasized in a broader sense, given that the cost of maintaining certain global networks based on peer-to-peer technology is starting to cause survival problems and requires optimizations that were already foreseen (Courtois et al. 2014). For example, Bitcoin power grid consumed approximately 2.55 GW of electricity in 2018, which is comparable to the consumption of countries, such as Ireland and its 3.1 GW (de Vries 2018). The hash rate, or the computing power needed to keep the network stable and the technology moving forward, is its main strength and weakness. The network will be more secure the higher the ratio is held, but more complex to mine and more computational and energy intensive. For example, some markets are currently affected by the COVID-19 pandemic, among other reasons. Of these, and in direct relation to cryptocurrencies, we must highlight the lack of stock and increase in computer components (mostly graphic cards) used for mining algorithms (Allan 2021; Faulkner 2021). We have recently seen mining farms using laptops in parallel due to their lower power consumption or companies, such as Nvidia Corporation (2021), launching versions exclusively for these purposes. The use of these technologies is promising but uncertain based on their overall cost alone (Li et al. 2019).

Should these tools be separated generally from the technology created at the level of future research?

As mentioned before, a constant relationship has both a technological and an economic side. Undoubtedly, the impact of technology and its multiple applications will keep them together, so this separation will not materialize. Although cryptocurrencies have led the path, as shown in Fig. 15, blockchain technology is the main topic that will eventually leave Bitcoin and Ethereum as basic or niche topics, as Shen et al. (2020) concluded.

Can the technology created be applied to more business issues, and can they benefit from it?

Above all, the Blockchain network is the pioneering technology that has appeared in a number of publications on cryptocurrencies (Yli-Huumo et al. 2016). Since 2016, several authors, such as Yu Zhang, Young-Sik Jeong, K.K.R. Choo or J.H. Park have established this trend, with the highest number of mentions of blockchain appearing in late 2020. Blockchain is a disruptive technology that can be used in all subject areas. This multiplicity of uses made necessary a systematic review, with special attention to business and economics (Xu et al. 2019). This suggests that we should take into account the application of the base technology and its potential applications at the business level (Zhao et al. 2016). Moreover, the cryptocurrency technology should be considered.

Based on the blockchain network analysis, this technology has great potential and offers many opportunities for the business area (Xu et al. 2019). The blockchain encryption system allows, for example, conducting secure and reliable financial transactions quickly, thanks to the distribution on independent nodes. The system also makes the data more difficult to falsify since it must be exchanged from multiple nodes simultaneously and allows the realization of smart contracts. Furthermore, it keeps the information more accessible because, as long as a node is still online, the information can be accessed; it does not have a single source server (Felin and Lakhani 2018; Gatteschi et al. 2018; Tönniseen et al. 2018; Chang et al. 2019).

## Conclusion

This study has reviewed an 8-year international search related to cryptocurrency due to bibliometric analysis of the WoS and Scopus databases.

The results show the positive evolution both in terms of the number of articles published and citations, with a growing number of publications and relevance in recent years. Comparing the evolution of both databases, we determine that WoS contains a greater number of citations received, whereas the Scopus database includes a greater number of articles. The main topics or research areas that contain the concepts related to cryptocurrencies are computer science and economics. If we delve further into the number of research areas in both databases, limiting the criteria to articles only, the enormous amount of categorical division seems to indicate that it is an interdisciplinary branch. However, on closer inspection, this perception changes because the majority of knowledge areas are related to the aforementioned sciences (i.e., computer science and economics). The subsequent thematic areas are legal sciences, criminology, philosophy, and physics.

The countries with the greatest number of publications are the USA, UK, and China, with the latter appearing alongside Canada in analyzing the most relevant keywords. The constant evolution of the regulatory framework regarding cryptocurrencies has generated various controversies at a global level. One notable case is in China, where after the general ban on Bitcoin trading in 2017, the Hangzhou Internet Court recently granted it a new status as a virtual asset. Hangzhou Internet Court was responsible for making cryptocurrencies public and reversing the ban without being considered fiat money. Meanwhile, the most used language for communications is English, coinciding with the native language of two of the countries with the highest rate of published articles. In contrast, although Chinese is not the language with the highest number of publications, China is one of the most often recurrent keywords in the last three years, making it a country showing the most interest in the subject. The authors’ cluster analysis also demonstrates the high participation rate they acquire.

A more in-depth analysis confirms that the main journals and authors belonging to the ranking also belong to the countries with the highest number of publications, to clarify any doubts that may arise from this new phenomenon. The number of outstanding journals and authors is increasing, but note that, especially when referring to authors, the wide participation of the Asian continent is prevalent if Scopus references are taken into account and even if the journals are English-speaking.

From the keywords obtained from the documents, the most frequent topics in the world of cryptocurrencies can be linked and recognized. Although WoS mainly contains words related to Bitcoin, Blockchain, and the volatility of these cryptocurrencies, Scopus publications focus on Bitcoin, Blockchain, and the technological aspects derived from them. Due to the importance of Blockchain technology, the publications on this topic have doubled in the last two years. A basic analysis of the theme shows a total of 550 articles in 2018, whereas the figure exceeds 1100 2019 in WoS. Scopus in turn shows results of approximately 650 and 1370. This is evidence of new lines of research among which stand out, blockchain appearing on both platforms as noteworthy, and Smart Contracts as an alternative to the conclusion of classic contracts that had been conducted.

At this point, and after starting to look at the reviews, especially of the most important keywords or the evolution in the discussion, we can see how the theory and background of cryptocurrencies has begun to conclude the publications on cryptocurrencies, leaving practical research as a new line of research. This opens the way to other interdisciplinary studies, especially after the controversy over the lack of regularization and harmonization in matters, such as legislative issues. Internally, these currencies are constantly revising and evolving to rectify the problems they previously had. Thus, the current information about them will be transformed by version periods, closing the chapter on version 1.0 and analyzing the modifications corresponding to version 2.0.

Finally, despite this study’s contribution, it also has some limitations. First, the field of study is based solely on two of the most influential academic databases (WoS and Scopus). Second, the type of document included in the analysis has been limited to articles. Given the recent creation of the topic and trying to cover the largest possible field of study, expanding the results with Google Scholar as a third data source or using a wide range of publication types could yield a larger document count, which in turn could change the results, especially concerning the keywords used. If the subject were focused on documents from Google Scholar, but the type of publication was not delimited, some 7750 total documents would be obtained. The following will be included in the top 10 publications: “Blockchain technology: Beyond bitcoin,” followed by “Zerocash: Decentralized anonymous payments from bitcoin,” and “The inefficiency of Bitcoin.” Although in different positions, all these articles are well placed in the two databases considered in this study. However, if the document type were to be extended, the existing procedural paper with the same time limitation as the articles in WoS amounts to 875, which, together with 684 articles, would add up to a total of 1559 of the 1678 results obtained. Scopus would yield a total of 1281 and 771, respectively, showing that 83.2% of the 2467 total results without applying filters are of both classes. In this way, an analysis of almost all the elements could be conducted.

## Data Availability

The datasets analyzed during the current study are available on the following websites: Price: https://coinmarketcap.com, Web of Science: http://wos.fecyt.es/, Scopus: https://www.scopus.com/

## Abbreviations

A: Articles
AC: Average citation
Au: Authors
BTC: Bitcoin
C: Country
ETH: Ether
FP: First publication
H: H-Index
J: Journals
JCR: Journal citation reports
LP: Last publication
R: Position in the ranking
RC: Research area scopus
RW: Research area WoS
Sco: SCOPUS
SJR: Scimago Journal Rank
TC: Total cites
WoS: Web of Science
